# Translation and validation of the Urdu version of the European organization for research and treatment of cancer core quality of life questionnaire (EORTC QLQ-C30) and brain module (QLQ-BN20) in primary brain tumor patients

**DOI:** 10.1186/s41687-021-00354-6

**Published:** 2021-09-06

**Authors:** Nida Zahid, Russell Seth Martins, Wajeeha Zahid, Wardah Khalid, Iqbal Azam, Shireen Shehzad Bhamani, Nargis Asad, Khabir Ahmad, Adnan Abdul Jabbar, Muhammad Shahzad Shamim, Rashid Jooma Khan, Gohar Javed, Ehsan Bari, Syed Ather Enam

**Affiliations:** 1 Department of Surgery, Aga Khan University Hospital, Stadium Road, Karachi, 74800 Pakistan; 2 Medical College, Aga Khan University Hospital, Stadium Road, Karachi, 74800 Pakistan; 3 Department of Community Health Sciences, Aga Khan University Hospital, Stadium Road, Karachi, 74800 Pakistan; 4 School of Nursing and Midwifery, Aga Khan University Hospital, Stadium Road, Karachi, 74800 Pakistan; 5 Department of Psychiatry, Aga Khan University Hospital, Stadium Road, Karachi, 74800 Pakistan; 6 Department of Oncology, Aga Khan University Hospital, Stadium Road, Karachi, 74800 Pakistan

**Keywords:** Primary brain tumor, Quality of life, Validity, Reliability, Resilience, Depression, Correlation, Urdu

## Abstract

**Introduction:**

This study translated and validated the Urdu version of the European Organization for Research and Treatment of Cancer Quality of Life (QoL) Questionnaire (EORTC QLQ-C30) and Brain Module (QLQ-BN20) amongst patients with primary brain tumors (PBT) in Pakistan, and assessed the correlation of QoL with resilience, depression, and anxiety.

**Methods:**

Translation of the EORTC QLQ-C30 and QLQ-BN20 was performed as per EORTC guidelines. A survey comprising of Urdu translations of EORTC QLQ-C30, QLQ-BN20, Wagnild and Young Resilience Scale (RS-14) and Hospital Anxiety and Depression Scale was administered to patients with PBT at a tertiary care hospital in Pakistan. Reliability (via Cronbach alpha), content validity index (CVI) scores, construct validity, and inter-scale correlations were assessed.

**Results:**

Our sample consisted of 250 patients with PBT, most commonly glioma (46.8%) and meningioma (21.2%). All patients were able to understand the Urdu translations. The Cronbach alphas for the QLQ-C30 and the QLQ-BN20 were 0.860 and 0.880, respectively. The CVI scores for clarity and relevance were high for both the EORTC QLQ-C30 (0.98 and 0.96, respectively) and the QLQ-BN20 tool (0.81 and 0.95, respectively). The global QoL domain (EORTC QLQ-C30) showed significant positive correlations with resilience (r = 0.422), and significant negative correlations with depression (r =  − 0.541) and anxiety (r =  − 0.502). Strong inter-scale correlations were observed between physical functioning and insomnia (r =  − 0.690) and role functioning and insomnia (r =  − 0.641).

**Conclusion:**

Our study confirms the Urdu versions of the EORTC QLQ-C30 and QLQ-BN20 as valid clinical tools for the measurement of QoL in primary brain tumors patients within the cultural and socioeconomic context of Pakistan.

**Supplementary Information:**

The online version contains supplementary material available at 10.1186/s41687-021-00354-6.

## Plain language summary

Quality of life (QoL) is an important facet of well-being for patients with primary brain tumors (PBTs), as these individuals face significant distress during the course of their illness and treatment. It is important to have valid and reliable tools to accurately measure the QoL of patients with PBTs. The EORTC QLQ-C30 (European Organization for Research and Treatment of Cancer Quality of Life Questionnaire) and its brain tumor-specific module EORTC QLQ-BN20 (EORTC QLQ-Brain Neoplasms 20) are exactly that.

However, the use of the EORTC QLQ-C30 and QLQ-BN20 is limited in Pakistan, where the national language is Urdu, and the majority of patients are of low socioeconomic backgrounds. Since no Urdu translations of the EORTC QLQ-C30 and QLQ-BN20 exist, we aimed to translate and validate these tools to enable their applicability in Pakistan.

The Urdu versions of the EORTC QLQ-C30 and QLQ-BN20 demonstrated good validity amongst patients with PBTs. Thus, our study confirms the EORTC QLQ-C30 and QLQ-BN20 as valuable clinical tools for the measurement of QoL in primary brain tumors patients within the linguistic, cultural and socioeconomic context of Pakistan.

**Supplementary Information:**

The online version contains supplementary material available at 10.1186/s41687-021-00354-6.

## Introduction

Malignant primary brain tumors (PBTs) are responsible for 2.7% of cancer deaths worldwide [1]. In the United States of America (USA), the incidence rate of PBTs is approximately 14.8/100,000/year, and morality rate is greater in males (5.6/100,000) as compared to females (3.7/100,000) [2]. In developing countries, however, both the incidence and mortality rates of PBTs is lower than those seen in developed countries [2]. In Pakistan, which is a lower-middle-income country (LMIC) in South Asia, malignant PBTs comprise around 3.6% of all malignancies [3]. However, the levels of distress experienced by patients with malignant PBTs is higher than that experienced by patients suffering from most other types of malignancies [4].

Quality of life (QoL) is a broad, multi-faceted concept that encompasses functionality and well-being in the physical, emotional, and psychosocial domains [5]. It is an increasingly important outcome in clinical neuro-oncology [5]. The vast majority of patients with PBTs face varying levels of physical, emotional, or cognitive distress. This distress may be attributed to factors such as physical disability, disfigurement, sensorimotor deficits, losses of individual freedoms, employment, and income, and social stigma [4]. Moreover, mental health outcomes, particularly depression, are strongly associated with poorer QoL in patients with PBTs [6]. Resilience, which is the capacity of individuals to maintain stable physical and cognitive functionality despite the many challenges of cancer, may help protect against adverse mental health outcomes and improve QoL in patients with PBTs [7–9]. Amongst the several tools designed to assess QoL amongst patients with PBT, the EORTC QLQ-C30 (European Organization for Research and Treatment of Cancer Quality of Life Questionnaire) and its brain tumor-specific module EORTC QLQ-BN20 (EORTC QLQ-Brain Neoplasms 20) have proved to be brief, reliable, and valid assessment measures [10, 11].

Differences in languages and cultures across the world have led to the translation of the EORTC QLQ-C30 and QLQ-BN20 into many different languages. The EORTC QLQ-C30 has been used in over 3000 studies to date and has been translated and approved in over 100 languages [12]. Although this includes Urdu, the national and official language of Pakistan, the validation of the Urdu version of the EORTC QLQ-C30 in a Pakistani population has to the best of our knowledge only been carried out in a cohort of 70 patients with hematologic malignancies [13]. The EORTC QLQ-BN20 has been less widely translated and validated, and never before in Urdu in a Pakistani population of patients with PBT [12]. There is an increasing need to assess the QoL experienced by patients with PBTs in Pakistan using proven tools such as the EORTC QLQ-C30 and QLQ-BN20. Due to cultural differences and a sizeable percentage of patients with PBT being from lower socioeconomic and less educated backgrounds, the English versions of the EORTC QLQ-C30 and QLQ-BN20 are of limited utility in a Pakistani setting. Moreover, although more than 10 different languages are spoken by the different cultural groups and ethnicities in Pakistan, Urdu is spoken and understood throughout the country. Thus, this study aimed to formulate and validate an appropriately translated Urdu version of the EORTC QLQ-C30 and QLQ-BN20 amongst patients with PBT in Pakistan. In addition, this study also aimed to assess the correlation of QoL with resilience, depression, and anxiety.

## Methods

### Study tools

The two tools validated in this study were the European Organization for Research and Treatment of Cancer Quality of Life Questionnaire (EORTC QLQ-C30) and the EORTC QLQ-Brain Neoplasms 20 (EORTC QLQ-BN20). Permission was obtained from the EORTC for the translation and validation of both tools. In addition, to explore the correlation of QoL with resilience, depression, and anxiety, two additional tools were included in the survey: Wagnild and Young’s Resilience Scale (RS-14) and Hospital Anxiety and Depression Scale (HADS). Thus, the final survey instrument consisted of a section on demographic and clinical characteristics, followed by the EORTC QLQ-C30, EORTC QLQ-BN20, RS-14 and HADS:

- *EORTC QLQ-C30*: A 30-item QoL measure for patients with cancer. The tool comprises five multi-item functional scales (physical, role, cognitive, emotional, and social), three symptom scales (fatigue, pain, and nausea and vomiting), a global health and QoL scale, and single items for measurement of other symptoms frequently experienced by cancer patients (such as dyspnea, appetite loss, sleep disturbance, constipation, and diarrhea), in addition to the perceived financial impact of the disease and treatment [10]. All items are scored using a 4-point Likert scale (1: ‘not at all’; to 4: ‘very much’), except for two items in the global health/QoL scale which instead employ modified 7-point linear analog scales [14]. The functioning and global QoL subscales are scored ranging from 0 to 100, where higher scores imply favorable conditions. However, while symptom subscales are also scored ranging from 0 to 100, higher scores in these subscales imply greater symptoms i.e., unfavorable conditions.
- *EORTC QLQ-BN20*: A 20-item QoL measure specifically for patients with primary brain neoplasms [11]. The tool comprises four domains all relevant to the disease (future uncertainty, visual disorder, motor dysfunction, and communication deficit), in addition to seven single items (headaches, seizures, drowsiness, hair loss, itchy skin, weakness of legs, bladder control). All items are scored using a 4-point Likert scale (1: ‘not at all’; to 4: ‘very much’) and are then linearly converted to a 0–100 scale, where a higher score implies unfavorable conditions.
- *RS-14*: A 14-item measure of five core characteristics of resilience (purposeful life, perseverance, equanimity, self-reliance and existential loneliness) that uses a 7-point Likert Scale to calculate an aggregate score for resilience [15]. The higher the score on the RS-14, the higher the resilience. The validated Urdu version of RS-14 was used, which has an acceptable Cronbach’s alpha of 0.763 [16]. We re-verified the internal consistency of the translated RS-14 and found a Cronbach’s alpha of 0.903, demonstrating excellent internal consistency of the RS-14 in the current population of brain tumor patients.
- *Hospital Anxiety and Depression Scale (HADS)*: A 14-item tool using a 4-point ordinal scale to measure depression and anxiety. The lower the score on the HADS, the more favorable the outcome. The Urdu version of the HADS [17] was used. The Urdu version of the HADS has been validated in pregnant females, and has been found to have an overall Cronbach’s alpha of 0.84 [18]. The Cronbach’s alpha for the depression and anxiety subscales were 0.64 and 0.82, respectively [18]. We re-evaluated the internal consistency for our sample. While the tool's overall internal consistency (Cronbach’s alpha: 0.89) and that of the anxiety subscale (Cronbach’s alpha: 0.81) were comparable to the previously reported values, the Cronbach’s alpha for the depression scale amongst our sample (Cronbach’s alpha: 0.86) was higher than previously reported.

### Translation of study tools and pilot testing

For purpose of validation in Urdu, the EORTC QLQ-C30 and QLQ-BN20 underwent a translation process in accordance with EORTC standards [19] and COSMIN Study Design Checklist for Patient-reported Outcome Measure Instruments [20]. Two translators initially translated the English versions of the EORTC QLQ-C30 and QLQ-BN20 into Urdu independently. Both translators involved in the forward translation were bilingual, with native proficiency in Urdu and full professional proficiency in English, and with more than 7 years of experience in translation of healthcare-related surveys. To reduce the risks of bias and to identify subtle discrepancies, one of the translators was aware of the constructs that the tools were intended to measure, while the other translator was naïve to the intended purpose of the tools. One consolidated Urdu version was then produced, which subsequently underwent backwards translation to English by two translators independently. Both translators involved in the backwards translation were native Urdu speakers with full professional proficiency in English, and were naïve to the intended purpose of the tools. A single consolidated backwards-translated English version of each tool was created and reviewed by the research team for consistency with the original English tools. Differences in the translation were then reviewed and settled in the presence of a third independent translator who was aware of the constructs measured by the tools. The difficulties encountered during the translation process are described in the Additional files 1 and 2. The preliminary Urdu translated versions were pilot tested on 25 (i.e., 10% of calculated minimum required sample size) brain tumor patients who were native Urdu speakers. This pilot testing, a form of linguistic validation of the comprehensibility of the tools, was performed in accordance with the guidelines published by the EORTC [21]. Prior to the administration of the tool via individual interviews, patients participating in the pilot testing were instructed to comment on whether any questions in the tool were difficult to understand, difficult to answer, confusing, upsetting, or offensive. Patients were also asked to rate the comprehensibility of each item in the tools using a Likert scale of 1–4 (see “Content validity index”). No major areas of improvement were identified by participants during the pilot testing, and only a few minor revisions were effected to produce a final Urdu translation of both tools. These final forms were included in the survey instrument of the current study.

### Study setting

This survey was conducted over the period November 2019 to May 2020 at the Aga Khan University Hospital (AKUH), which is a Joint Commission International Accreditation (JCIA-accredited) hospital in Karachi, Pakistan.

The institutional review board of AKUH granted ethical approval for this study (Reference Number: 5154-Sur-ERC-17). The complete protocol of this study has been published by the authors [8].

### Study subjects and sampling

Our target population was adult (≥ 18 years) patients treated for primary malignant brain tumors at AKUH. Patients were included if they were currently ≥ 4 weeks post-initiation of treatment, provided written informed consent for participation, and were residing in Pakistan for at least the past 3 months. The lattermost criterion was to ensure the validation of the EORTC QLQ-C30 and QLQ-B20 tool was achieved while measuring QoL in the context and setting of Pakistan, as patients residing abroad may experience QoL different to their counterparts residing in Pakistan.

Exclusion criteria included patients with history of psychiatric illness or on prescription psychiatric medications, or with debilitating comorbidities such as stroke or renal failure. However, patients with comorbid hypertension (HTN), type 2 diabetes mellitus (T2DM) or chronic obstructive pulmonary disease (COPD) were not excluded from the sample, as the prevalence of these comorbidities is high amongst the Pakistani population [22]. Moreover, these comorbidities are commonly seen in patients with brain tumors [23]. Thus, including such patients ensured a representative population.

Non-probability consecutive sampling was used for recruiting participants. Trained research assistants approached brain tumor patients visiting AKUH as per their scheduled appointments at the surgical/oncology clinics. Potential participants were screened for eligibility by the research assistants. After providing their informed consent, they were administered the EORTC QLQ-C30 and QLQ-BN20 tools as part of a survey conducted as an interview by the research assistants. Although both the EORTC QLQ-C30 and QLQ-BN20 tools are generally self-administered, we opted to routinely administer them via interviews so as to include patients within our setting who lacked the literacy to read (similar to Montazeri et al. in their Iranian translation and administration [24]). A minimum sample size of 250 was required. This was calculated using the one population mean formula, a standard deviation (SD) of 20, 5% level of significance with precision of 2.5, and by adjusting the sample size for 10% rate of incomplete responses [8].

### Content validity index

Content validation of the EORTC QLQ-C30 and QLQ-BN20 tool was performed with accordance to the COSMIN Study Design Checklist for Patient-reported Outcome Measure Instruments [20]. Five experts (a psychologist, epidemiologist, biostatistician, neurosurgeon, and mental health researcher) were asked to rate the relevance of all items of both tools using a Likert scale of 1–4. In addition, patients participating in the pilot testing were requested to rate the relevance and clarity (comprehensibility) of each item in the tools using a Likert scale of 1–4. However, the comprehensiveness of EORTC QLQ-C30 and QLQ-BN20 was not assessed from the professionals’ or patients’ perspective. Both tools used were pre-constructed and have been used in their current form in studies globally, and the comprehensiveness of their content is well established. Content validity index (CVI) scores for clarity and relevance of the tools were calculated as described below in the Statistical Analysis section.

### Statistical analysis

Data was analyzed using SPSS Statistics for Windows version 23.0 (IBM Corp., Armonk, N.Y., USA). Two members of the research team were involved in data analysis. Categorical variables were represented as frequencies and percentages. Numerical variables were represented by mean and standard deviation or median and interquartile range. The Pearson correlation coefficients were calculated to determine the construct validity between EORCT QLQ-C30 and QLQ-BN20 with RS-14 and HADS. Reliability was estimated using the Cronbach’s alpha coefficient, with a value ≥ 0.70 considered acceptable. Content validation index (CVI) was reported to determine the relevance and clarity of the content of the tool. For each item, the score suggested by the raters was summed up and divided by the total number of raters to obtain an average score for each item. The sum of the average score of each item was further divided by the total number of items to obtain a CVI score ranging from 0 to 1 (1 = perfect agreement; and 0 = no agreement). Interscale correlations were also calculated for both the EORCT QLQ-C30 and QLQ-BN20. A *p* value of < 0.05 was considered significant.

## Results

### Sample characteristics

A total of 255 patients with PBTs were approached for inclusion in this survey. However, five amongst these were ineligible (1 patient no longer resided in Pakistan and 4 patients had pre-existing psychiatric disorders), leaving a total of 250 participants in the final sample. Their mean age was 44 ± 0.83 years, and 68% were male. The most common mother tongue was Urdu (30.8%), with others including Sindhi (18.8%), Punjabi (14.8%) and Pushto (10.4%). However, every patient was able to understand and fluently converse in Urdu, with no language barriers to administering the survey. The diagnoses of PBTs included glioma (46.8%) and meningioma (21.2%). Most participants had undergone tumor biopsy (78%). 9.6% patients reported receiving radiotherapy, 4.4% chemotherapy, and 25.2% combination therapy, while 60.8% reported no adjuvant therapy (Table 1).

**Table 1:** Participant demographic characteristics and disease-related factors

Variables	N = 250 N (%)/Median (IQR)
Age (years)	42 (33–54)
*Gender*	
Male	169 (67.6)
Female	81 (32.4)
*Formal schooling*	
Yes	229 (91.6)
No	21 (8.4)
*Informal schooling*	
Yes	44 (17.6)
No	206 (82.4)
*Mother tongue*	
Urdu	77 (30.8)
Sindhi	47 (18.8)
Punjabi	37 (14.8)
Pushto	26 (10.4)
Saraiki	11 (4.4)
Balochi	10 (4.0)
Hindko	6 (2.4)
Other	36 (14.4)
*Marital status*	
Married	205 (82.0)
Single	37 (14.8)
Other	8 (3.2)
*Monthly household income (PKR/USD)*	
No income	18 (7.2)
1000–25,000 ($6.04–$151)	40 (16.0)
25,000–40,000 ($151–$242)	26 (10.4)
40,000–80,000 ($242–$484)	69 (27.6)
80,000–170,000 ($484–$1028)	97 (38.8)
*Tumor type*	
Glioma	117 (46.8)
Meningioma	53 (21.2)
Schwannoma	12 (4.8)
Pituitary	44 (17.6)
Others	24 (9.6)
*Surgical intervention*	
Only biopsy	195 (78.0)
Only total resection	11 (4.4)
Multiple interventions	27 (10.8)
No surgical intervention	17 (6.8)
*Adjuvant therapy*	
Chemotherapy	11 (4.4)
Radiotherapy	24 (9.6)
Combination	63 (25.2)
No adjuvant therapy	152 (60.8)
*Treatment stage for brain tumor*	
On-going	138 (55.2)
Complete	112 (44.8)

PKR, Pakistani Rupee; USD, US Dollars

### Internal consistency or reliability

Table 2 depicts the reliability of the EORTC QLQ-C30 tool and QLQ-BN20 tool. The Cronbach alphas for the 30 items of the QLQ-C30 and the 20 items of the QLQ-BN20 were 0.860 and 0.880, respectively, indicating good internal consistency of both tools. The internal consistency for the global health status scale of the QLQ-C30 was also good (Cronbach’s alpha = 0.800; *p* < 0.001). The Cronbach’s alpha for the 15 items of the functional scale of the QLQ-30 tool was 0.74 (range: 0.630–0.830), indicating acceptable to good internal consistency (*p* < 0.001). The overall internal consistency for the 11 items of the symptom scale of the QLQ-C30 was also good (Cronbach’s alpha 9 = 0.82 (range: 0.800–0.86); *p* < 0.001). Lastly, the consistency of the four domains of the QLQ-BN20 demonstrated acceptable-to-good consistency (Cronbach’s alpha range: 0.703–0.868).

**Table 2:** Internal consistency of EORTC QLQ-C30 (1A) and QLQ-BN20 (1B)

1A: QLQ-C30	
	Cronbach's alpha (*p* value)
Overall	0.86 (< 0.001*)
*Global*	
Global status	0.80 (< 0.001*)
*Functional*	
Physical functioning	0.83 (< 0.001*)
Role functioning	0.82 (< 0.001*)
Emotional functioning	0.75 (< 0.001*)
Cognitive functioning	0.63 (< 0.001*)
Social functioning	0.79 (< 0.001*)
*Symptoms*	
Fatigue	0.36 (< 0.001*)
Nausea and vomiting	0.40 (< 0.001*)
Pain	0.46 (< 0.001*)

1B: QLQ-BN20	
Overall	0.88 (< 0.001)*
Future uncertainty	0.801 (< 0.001*)
*Symptoms*	
Visual disorder	0.790 (< 0.001*)
Motor dysfunction	0.703 (< 0.001*)
Communication deficit	0.868 (< 0.001*)

*Significant at *p*-value < 0.05 by reliability analysis

### Content validity

The expert-reported CVI scores for relevance of the Urdu version of the EORTC QLQ-C30 and QLQ-BN20 tool were 0.96 and 0.95, respectively, indicating excellent agreement among the five experts. The patient-reported CVI scores for clarity and relevance of the Urdu version of the EORTC QLQ-C30 were 0.92 and 0.93, respectively. Similarly, the patient-reported CVI scores for clarity and relevance of the Urdu version of the QLQ-BN20 tool were 0.80 and 0.93, respectively. These results indicate good-to-excellent agreement among the 25 patients for clarity and relevance of both tools.

### Construct validity

The correlation of QoL (as measured by EORTC QLQ-C30 and QLQ-BN20) with resilience (RS-14) was assessed using Pearson correlation coefficients (Tables 3, 4). We observed a significant moderate positive correlation between global status of EORTC QLQ-C30 and resilience (r = 0.422; *p* value < 0.001). Similarly, there was a significant moderate positive correlation between the 5 domains of the functional scale of EORTC QLQ-C30 and resilience (r ranging from 0.462 to 0.570; *p* value < 0.001). Lastly, there was a significant moderate negative correlation between future uncertainty and resilience (r = −0.473, *p* < 0.001).

**Table 3:** Correlation between QLQ-C30 with resilience, depression and anxiety (construct validity)

QLQ C-30	Resilience score	Depression score	Anxiety score
	r (*p* value)		
Global quality of life	0.422 (< 0.001)*	− 0.541 (< 0.001)*	− 0.502 (< 0.001)*
Functional			
Physical functioning	0.570 (< 0.001)*	− 0.545 (< 0.001)*	− 0.276 (< 0.001)*
Role functioning	0.472 (< 0.001)*	− 0.542 (< 0.001)*	− 0.320 (< 0.001)*
Emotional functioning	0.462 (< 0.001)*	− 0.688 (< 0.001)*	− 0.704 (< 0.001)*
Cognitive functioning	0.504 (< 0.001)*	− 0.467 (< 0.001)*	− 0.293 (< 0.001)*
Social functioning	0.478 (< 0.001)*	− 0.490 (< 0.001)*	− 0.319 (< 0.001)*
Symptoms			
Fatigue	− 0.075 (0.236)	0.298 (< 0.001)*	0.327 (< 0.001)*
Nausea and vomiting	− 0.158 (0.012)*	0.243 (< 0.001)*	0.275 (< 0.001)*
Pain	− 0.191 (0.002)*	0.412 (< 0.001)*	0.433 (< 0.001)*
Dyspnea	− 0.172 (0.006)*	0.334 (< 0.001)*	0.332 (< 0.001)*
Insomnia	− 0.508 (< 0.001)*	0.463 (< 0.001)*	0.267 (< 0.001)*
Appetite loss	− 0.305 (< 0.001)*	0.554 (< 0.001)*	0.500 (< 0.001)*
Constipation	− 0.179 (0.004)*	0.119 (0.061)	0.071 (0.261)
Diarrhea	− 0.050 (0.429)	0.162 (0.01)*	0.036 (0.576)
Financial difficulties	− 0.314 (< 0.001)*	− 0.266 (< 0.001)*	0.186 (0.003)*

r = Pearson correlation coefficient

*Significant at *p* value < 0.05

**Table 4:** Correlation between QLQ-BN20 with resilience, depression and anxiety (construct validity)

QLQ-BN20	Resilience score	Depression score	Anxiety score
Future uncertainty	− 0.473 (< 0.001)*	0.614 (< 0.001)*	0.514 (< 0.001)*
*Symptoms*			
Visual disorder	− 0.347 (< 0.001)*	0.277 (< 0.001)*	0.139 (0.029)*
Motor dysfunction	− 0.571 (< 0.001)*	0.458 (< 0.001)*	0.209 (0.001)*
Communication deficit	− 0.514 (< 0.001)*	0.425 (< 0.001)*	0.211 (0.001)*
Headache	− 0.121 (0.054)	0.236 (< 0.001)*	0.246 (< 0.001)*
Seizures	− 0.168 (0.008)*	0.149 (0.018)*	0.014 (0.822)
Drowsiness	− 0.408 (< 0.001)*	0.442 (< 0.001)*	0.262 (< 0.001)*
Hair loss	− 0.196 (0.002)*	0.144 (0.022)*	0.138 (0.029)*
Itchy skin	− 0.166 (0.008) *	0.185 (0.003)*	0.145 (0.022)*
Weakness in leg	− 0.478 (< 0.001)*	0.437 (< 0.001)*	0.284 (< 0.001)*
Bladder control	− 0.218 (0.001)*	0.277 (< 0.001)*	0.220 (< 0.001)*

r = Pearson correlation coefficient

*Significant at *p* value < 0.05

When assessing the correlation between the EORCT QLQ-C30 and the QLQ-BN20 tool with depression and anxiety as measured using the HADS, we observed a significant moderate negative correlation between global status of EORTC QLQ-C30 with depression (r = −0.541; *p* < 0.001) and anxiety (r = −0.502; *p* < 0.001). Additionally, there was a significant moderate-to-strong negative correlation between the 5 domains of the functional scales of the EORTC QLQ-C30 i.e., with both depression (r ranging from −0.467 to −0.688; *p* < 0.001) and anxiety (r ranging from −0.276 to −0.704; *p* < 0.001). There was a strong positive correlation between future uncertainty and depression (r = 0.614, *p* < 0.001) and moderate correlation between future uncertainty and anxiety (r = 0.514, *p* < 0.001). The Pearson’s correlation coefficients for the EORTC QLQ-C30 and QLQ-BN20 with the HADS are shown in Tables 3 and 4, respectively.

### Inter-scale correlation of EORTC QLQ-C30 and EORTC QLQ-BN20

When assessing inter-scale correlations of EORTC QLQ-30, we observed a significant weak-to-moderate positive correlation between global QoL and the 5 functional domains (r ranging from 0.384 to 0.561; *p* < 0.001). We observed a significant moderate-to-strong positive correlation (r ranging from 0.40 to 0.68; *p* < 0.01) within the 5 functional domains of EORCT QLQ-30, with strongest correlation between physical and role functioning (r = 0.600; *p* < 0.001), physical and social functioning (r = 0.631; p=0.001), role and social functioning (r = 0.680; *p* < 0.001).

Moreover, a significant weak-to-strong negative correlation was observed between 5 functional domains and 8 symptom domains of EORCT QLQ-30 (r ranging from −0.172 to −0.690; *p* < 0.01), with strongest correlations between physical functioning and insomnia (r = −0.690; *p* < 0.001) and role functioning and insomnia (r = −0.641; *p* < 0.001). In addition, within the symptom domains, the strong correlation was between fatigue and pain (r =0.602; *p* < 0.001).

When assessing inter-scale correlations of EORTC QLQ-BN20, we observed a significant weak-to-strong positive correlation of future uncertainty and 9 symptom domains (r ranging from 0.245 to 0.628; *p* < 0.001), with the strongest correlation between future uncertainty and drowsiness (r = 0.628; *p* < 0.001). The strongest correlations within the symptom domains were between motor dysfunction and drowsiness (r = 0.605; *p* < 0.001) and motor dysfunction and weakness in leg (r = 0.683; *p* < 0.001).

The interscale Pearson’s correlation coefficients for EORTC QLQ-C30 and QLQ-BN20 are shown in Tables 5 and 6, respectively.

**Table 5:** Interscale correlation of EORTC QLQ-C30

	GS	PF	RF	EF	CF	SF	F	NV	P	DY	I	AL	C	DI	FD
GS	1	0.384*	0.493*	0.561*	0.364*	0.436*	− 0.313*	− 0.297*	− 0.427*	− 0.139^+^	− 0.408*	− 0.454*	− 0.182*	− 0.038	− 0.429*
PF	0.384*	1	0.660*	0.472*	0.427*	0.631*	− 0.217*	− 0.208*	− 0.370*	− 0.207*	− 0.690*	− 0.409*	− 0.221*	− 0.065	− 0.324*
RF	0.493*	0.660*	1	0.461*	0.471*	0.680*	− 0.326*	− 0.322*	− 0.518*	− 0.265*	− 0.641*	− 0.486*	− 0.165*	− 0.172*	− 0.355*
EF	0.561*	0.472*	0.461*	1	0.440*	0.487*	− 0.435*	− 0.370*	− 0.505*	− 0.264*	− 0.422*	− 0.547*	− 0.166*	− 0.062	− 0.281*
CF	0.364*	0.427*	0.471*	0.440*	1	0.466*	− 0.200*	− 0.229*	− 0.332*	− 0.277*	− 0.498*	− 0.257*	− 0.275*	− 0.098	− 0.213*
SF	0.436*	0.631*	0.680*	0.487*	0.466*	1	− 0.265*	− 0.294*	− 0.381*	− 0.111*	− 0.589*	− 0.370*	− 0.201*	− 0.121	− 0.357*
F	− 0.313*	− 0.217*	− 0.326*	− 0.435*	− 0.200*	− 0.265*	1	0.399*	0.602*	0.248*	0.335*	0.412*	0.152^+^	0.132^+^	0.230*
NV	− 0.297*	− 0.208*	− 0.322*	− 0.327*	− 0.229*	− 0.294*	0.399*	1	0.326*	0.134^+^	0.259*	0.382*	0.471*	0.201*	0.241*
P	− 0.427*	− 0.370*	− 0.518*	− 0.505*	− 0.332*	− 0.381*	0.602*	0.326*	1	0.285*	0.426*	0.508*	0.215*	0.101	0.277*
DY	− 0.139^+^	− 0.207*	− 0.265*	− 0.264*	− 0.277*	− 0.111	0.248*	0.134^+^	0.285*	1	0.171*	0.248*	0.165*	0.267*	0.155^+^
I	− 0.408*	− 0.690*	− 0.641*	− 0.422*	− 0.498*	− 0.589*	0.335*	0.259*	0.426*	0.171*	1	0.350*	0.171*	0.096	0.367*
AL	− 0.454*	− 0.409*	− 0.486*	− 0.547*	− 0.257*	− 0.370*	0.412*	0.382*	0.508*	0.248*	0.350*	1	0.174*	0.214*	0.296*
C	− 0.182*	− 0.221*	− 0.165*	− 0.166*	− 0.275*	− 0.201*	0.152^+^	0.471*	0.215*	0.165*	0.171*	0.174*	1	0.075	0.147^+^
DI	− 0.038	− 0.061	− 0.172*	− 0.062	− 0.098	− 0.121	0.132^+^	0.201*	0.101	0.267*	0.096	0.214*	0.075	1	0.167*
FD	− 0.429*	− 324*	− 0.355*	− 0.281*	− 0.213	− 0.357*	0.230*	0.241*	0.277*	0.155^+^	0.367*	0.296*	0.147^+^	0.167*	1

GS, global scale; PF, physical functioning; RF, role functioning; EF, emotional functioning; CF, cognitive functioning; SF, social functioning; F, fatigue; NV, nausea and vomiting; P, pain; DY, dyspnea; I, insomnia; AL, appetite loss; C, constipation; DI, diarrhea; FD, financial difficulties

^+^Significant at *p* < 0.05

*Significant at *p* < 0.01

**Table 6:** Interscale correlations of EORCT QLQ-BN20

	FU	VD	M	CD	HA	SZ	DS	HL	IS	WL	BC
FU	1	0.402*	0.458*	0.346*	0.280*	0.346*	0.628*	0.245*	0.109	0.385*	0.358*
VD	0.402*	1	0.372*	0.467*	0.202*	0.167*	0.441*	0.216*	0.077	0.256*	0.288*
MD	0.458*	0.372*	1	0.568*	0.183*	0.191*	0.605*	0.203*	0.137^+^	0.683*	0.400*
CD	0.346*	0.467*	0.568*	1	0.092	0.141^+^	0.450*	0.046	0.235*	0.366*	0.187*
HA	0.280*	0.202*	0.183*	0.092	1	0.085	0.259*	0.134^+^	0.057	0.153^+^	(0.258*
SZ	0.346*	0.167*	0.191*	0.141^+^	0.085	1	0.254*	− 0.052	− 0.083	0.278*	0.024
DS	0.628*	0.441*	0.605*	0.450*	0.259*	0.254*	1	0.221*	0.176*	0.453*	0.348*
HL	0.245*	0.216*	0.203*	0.046	0.134	− 0.052	0.221*	1	0.325*	0.128^+^	0.485*
IS	0.109*	0.077	0.137^+^	0.235*	0.057	− 0.083	0.176*	0.176*	1	0.149^+^	0.242*
WL	0.385*	0.256*	0.683*	0.366*	0.153^+^	0.278*	0.453*	0.453*	0.149^+^	1	0.350*
BC	0.358*	0.288*	0.400*	0.187*	0.258*	0.024	0.348*	0.348*	0.242*	0.350*	1

FU, future uncertainty; VD, visual disorder; M, motor dysfunction; CD, communication deficit; HA, headache; SZ, seizures; DS, drowsiness; HL, hair loss; IS, itchy skin; WL, weakness in leg; BC, bladder control

^+^Significant at *p* < 0.05

*Significant at *p* < 0.01

## Discussion

Though health related QoL is an increasingly important outcome in the management of patients with malignant PBTs, it is challenging to measure QoL of patients with PBTs in Pakistan due to the lack of specific tools available in Urdu. In this regard, we attempted to translate and validate the EORTC QLQ-C30 and QLQ-BN20 in Urdu, the national and official language of Pakistan, to provide a valuable tool for the measurement of QoL of patients with PBTs in a clinical setting. With overall high internal consistency (Cronbach’s alpha: 0.86 and 0.88, respectively), and content validity index scores for patient-reported clarity (0.92 and 0.80, respectively) and expert-reported relevance (0.96 and 0.95, respectively), the Urdu versions of the EORTC QLQ-C30 and QLQ-BN20 tools displayed good validity in patients with PBTs in our study.

The overall experience of the translation and validation process of the EORTC QLQ-C30 and QLQ-BN20 in Urdu was relatively straightforward. The translations in Urdu were able to accurately convey the intended English equivalents, and none of the patients had any major issues with understanding the final Urdu version of either tool. This was encouraging, as the Urdu translations of both tools displayed high content validity for clarity and relevance. Moreover, as both tools were administered via interview as opposed to self-administration, our results also demonstrate the undiminished value of the EORTC QLQ-C30 and QLQ-BN20 tools in populations of LMICs where literacy may be low [24]. This study was conducted in Karachi, the largest city of the country, and home to all major ethnicities living in Pakistan. In addition, the Aga Khan University Hospital (AKUH) is one of the largest private quaternary care hospitals catering to diverse ethnic and socioeconomic groups within Karachi. The patients with PBTs in our sample represented heterogenous ethnicities and socioeconomic strata from within Pakistan, as evidenced by the diversity of mother tongues (Urdu: 30.8%, Sindhi: 18.8%, Punjabi: 14.8%, Pushto: 10.4%, Saraiki: 4.4%, Balochi 4%, and Hindko: 2.4%) and distribution of monthly family incomes. The diversity in patients’ demographics in our study strengthens the generalizability and utility of the Urdu translation of the EORTC QLQ-C30 and QLQ-BN20 as tools for measuring QoL within the sociocultural context of Pakistan.

The internal consistency of the Urdu version of the EORTC QLQ-C30 was high for the overall tool (Cronbach’s alpha: 0.86) and the global scale (0.80). The only other study reporting translation and validation of the EORTC QLQ-C30 in Urdu that we were able to find also reported a high Cronbach’s alpha of 0.82 for the overall tool [13].

Moreover, internal consistency was within minimum-to-ideal ranges (0.75–0.83) for most functional subscales in the current study. The exception was the subscale for cognitive functioning (0.63). The relatively low internal consistency of the cognitive functioning subscale has been observed in previous translation and validation studies in Korean (0.60) [25], Chinese (0.45) [26] and Japanese (0.63) [27], and also in validation studies of the original version of the EORTC QLQ-C30 by Aaronson et al. (0.54) [10], Bjordal et al. (0.28) [28], Osoba et al. (0.56) [29], and Ringdal et al. (0.65) [30]. Thus, our study also corroborates findings in existing literature which suggest that the cognitive functioning subscale of the EORTC QLQ-C30 has low reliability. This may be because memory and concentration are different aspects of cognitive functioning [26], although it is clinically more useful to measure cognitive functioning as a single construct. Lastly, in our sample, the consistency of the overall tool (0.88) and the four domains of symptoms in the QLQ-BN20 also demonstrated above minimum psychometric consistency (0.703–0.868). These ranges are similar to translation and validation studies conducted in other languages, including Persian (0.740–0.890) [31] and Chinese (0.753–0.869) [32]. Moreover, a review of the internal consistencies of the different translations of the EORTC QLQ-BN20 reviewed an overall range of internal consistency of the four subscales ranging from 0.71–0.90 [33]. Our results thus align with previous evidence and confirm the reliability of the Urdu version of the EORTC QLQ-BN20 in a Pakistani setting.

Lastly, inter-scale correlations for the Urdu version of the EORTC QLQ-C30 and QLQ-BN20 were significant in the expected directions, and similar to those seen previous studies validating the Iranian and Korean versions [24, 25].

The Urdu versions of both the EORTC QLQ-C30 and QLQ-BN20 tools also demonstrated acceptable construct validity, with the global quality of life domain showing significant positive correlations with resilience (r = 0.422), and significant negative correlations with depression (r = −0.541) and anxiety (r = −0.502). Previous studies have also reported similar positive correlations between QoL and resilience [34, 35] and similar negative correlations between QoL and depression and anxiety in cancer patients [36, 37]. In addition, the Hindi translation of the EORTC QLQ-BN20 has also demonstrated significant correlations with depression and anxiety as measured by HADS [38].

The Urdu translations of the EORTC QLQ-C30 and QLQ-BN20 hold considerable clinical relevance for the management of patients with malignant PBTs in Pakistan. Keeping in mind cultural differences in Pakistan and that most patients with PBT belong from less educated backgrounds, it is crucial to have tools that provide valid assessments of QoL in a Pakistani population. Urdu, being the national and official language, is spoken and understood throughout the country. Thus, our translation and validation of the EORTC QLQ-C30 and QLQ-BN20 provides a comprehensive, valid, and reliable method for the measurement of QoL in patients with malignant PBTs in Pakistan. Assessment of QoL may be incorporated into the routine management of patients with malignant PBTs in Pakistan, particularly in holistic goal setting, prognostication, and monitoring the impact of disease and therapy. In addition, the tools also provide a much-needed measure of patient-reported outcomes in clinical cancer research in Pakistan.

Our study has a few limitations. Firstly, we did not perform test-retest analysis to investigate stability. Additionally, the cross-sectional nature of the study does not capture changing relationships between QoL, resilience, depression, and anxiety. However, our study is the first validating the Urdu versions of EORTC QLQ-BN20 and QLQ-C30 in a sample of patients with malignant PBTs in Pakistan. Our results provides the preliminary base for further psychometric evaluation.

## Conclusion

Our study performed the translation of the EORTC QLQ-C30 and QLQ-BN20 to Urdu as per EORTC guidelines. The Urdu versions of the EORTC QLQ-C30 and QLQ-BN20 demonstrated good validity amongst patients with primary brain tumors. Thus, our study confirms the EORTC QLQ-C30 and QLQ-BN20 as valuable clinical tools for the measurement of QoL in primary brain tumors patients within the cultural and socioeconomic context of Pakistan.

## Supplementary Information

**Additional file 1**: Europeon Organization For Research and Treatment of Cancer (EORCT) Urdu Translation.

**Additional file 2**: Difficulties in Translation.

## Data Availability

The data that support the findings of this study are available from the corresponding author upon reasonable request.
