# Supplementary material for: Translation and validation of the Urdu version of the European organization for research and treatment of cancer core quality of life questionnaire (EORTC QLQ-C30) and brain module (QLQ-BN20) in primary brain tumor patients
Source: J Patient Rep Outcomes. 2021 Sep 6;5:79. doi: 10.1186/s41687-021-00354-6 (PMC8421474; doi:10.1186/s41687-021-00354-6)
Supplement: Supplementary file 1 — Additional file 1: Europeon Organization For Research and Treatment of Cancer (EORCT) Urdu Translation. [file 41687_2021_354_MOESM1_ESM.pdf]

EORTC QLQ-C30 (version 3) (General)(عمومی) (تیسری تبدیل شدہ دستاویز)

We are interested in some things about you and your health. Please answer all of the questions circling the number that best applies to you. There are no "right" or "wrong" answers. The information that you provide will remain strictly confidential

ہم آپ سے آپ کے اور آپکی صحت کے متعلق کچھ باتوں میں دلچسپی رکھتے ہیں۔ براہ مہربانی تمام سوالات کے جوابات، جو آپ سے سب سے مناسب سمجھتی ہوں، ان کے نمبر پر دائرہ لگا کر خود دیجئے۔ جوابات میں کوئی 'صحیح' یا 'غلط' نہیں۔ آپ کی دی گئی معلومات تمام ترصیغہ راز میں رہیں گی۔

S.NO	Items	Response
1.	Do you have any trouble doing strenuous activities, like carrying a heavy shopping bag or a suitcase?<br>کیا آپ کو محنت طلب سرگرمیوں میں دشواری پیش آئی۔ جیسا کہ بھاری وزن اٹھانا وغیرہ؟	1. Not at All بالکل بھی نہیں<br>2. A little تھوڑی سی<br>3. Quite a Bit کافی حد تک<br>4.Very Much بہت زیادہ
2.	Do you have any trouble taking a long walk?<br>***A long walk include e.g. walking to clinic from parking***<br>کیا آپ کو لمبی چہل قدمی میں دشواری ہوتی ہے؟	1. Not at Allبالکل بھی نہیں<br>2. A littleتھوڑی سی<br>3. Quite a Bitکافی حد تک<br>4.Very Much بہت زیادہ
3.	Do you have any trouble taking a short walk outside of the house?<br>***A short walk include going to nearby market or nearby park***<br>کیا آپ کو گھر سے باہر مختصر چہل قدمی میں کوئی تکلیف ہوتی ہے؟	1. Not at Allبالکل بھی نہیں<br>2. A littleتھوڑی سی<br>3. Quite a Bitکافی حد تک<br>4.Very Much بہت زیادہ
4.	Do you need to stay in bed or a chair during the day?<br>کیا آپ کو دن میں کرسی پر بیٹھنے یا بستر پر لیٹنے کی ضرورت پڑتی ہے؟	1. Not at Allبالکل بھی نہیں<br>2. A littleتھوڑی سی<br>3. Quite a Bitکافی حد تک<br>4.Very Much بہت زیادہ
5.	Do you need help with eating, dressing, washing yourself or using the toilet?<br>*Example: help in performing activities of daily living (ADL)*<br>کیا آپ کو کھانا کھانے، لباس تبدیل کرنے، منہ ہاتھ دھونے یا بیت الخلا استعمال کرنے میں کسی مدد کی ضرورت پڑتی ہے؟	1. Not at Allبالکل بھی نہیں<br>2. A littleتھوڑی سی<br>3. Quite a Bitکافی حد تک<br>4.Very Much بہت زیادہ

6.	Were you limited in doing either your work or other daily activities?<br>کیا آپ اپنے ذاتی کام کاج اور دیگر روز مرہ کیسر گرمیوں میں محدود ہو گئے ہیں؟	1. Not at Allبالکل بھی نہیں<br>2. A littleتھوڑی سی<br>3. Quite a Bitکافی حد تک<br>4.Very Much بہت زیادہ
7.	Were you limited in pursuing your hobbies or other leisure time activities such as reading a book, listening to music, gardening, going out?<br>کیا آپ کے فارغ اوقات کی سر گرمیاں محدود ہو گئی ہیں۔ جیسے کتاب پڑھنا، موسیقی سننا یا باغبانی وغیرہ؟	1. Not at Allبالکل بھی نہیں<br>2. A littleتھوڑی سی<br>3. Quite a Bitکافی حد تک<br>4.Very Much بہت زیادہ
8.	Were you short of breath?<br>کیا آپ کو سانس لینے میں مشکل ہوئی ہے؟	1. Not at Allبالکل بھی نہیں<br>2. A littleتھوڑی سی<br>3. Quite a Bitکافی حد تک<br>4.Very Much بہت زیادہ
9.	Have you had pain?<br>کیا آپ کو درد ہوا ہے؟	1. Not at Allبالکل بھی نہیں<br>2. A littleتھوڑی سی<br>3. Quite a Bitکافی حد تک<br>4.Very Much بہت زیادہ
10.	Did pain interfere with your daily activities?<br>کیا درد کی وجہ سے آپ کی روز مرہ کی سر گرمیاں متاثر ہوئی ہیں؟	1. Not at Allبالکل بھی نہیں<br>2. A littleتھوڑی سی<br>3. Quite a Bitکافی حد تک<br>4.Very Much بہت زیادہ
11.	Did you need to rest?<br>کیا آپ کو آرام کی ضرورت محسوس ہوئی؟	1. Not at Allبالکل بھی نہیں<br>2. A littleتھوڑی سی<br>3. Quite a Bitکافی حد تک<br>4.Very Much بہت زیادہ
12.	Have you had trouble sleeping?<br>کیا آپ کو سونے میں دشواری ہوئی ہے؟	1. Not at Allبالکل بھی نہیں<br>2. A littleتھوڑی سی<br>3. Quite a Bitکافی حد تک<br>4.Very Much بہت زیادہ
13.	Have you felt weak?<br>کیا آپ کو کمزوری محسوس ہوئی؟	1. Not at Allبالکل بھی نہیں<br>2. A littleتھوڑی سی<br>3. Quite a Bitکافی حد تک<br>4.Very Much بہت زیادہ

14.	Have you Lost appetite?<br>کیا آپ کی بھوک میں کمی آئی؟	1. Not at Allبالکل بھی نہیں<br>2. A littleتھوڑی سی<br>3. Quite a Bitکافی حد تک<br>4.Very Much بہت زیادہ
15.	Have you felt nauseated?<br>کیا آپ کو متلی محسوس ہوئی ہے؟	1. Not at Allبالکل بھی نہیں<br>2. A littleتھوڑی سی<br>3. Quite a Bitکافی حد تک<br>4.Very Much بہت زیادہ
16.	Have you vomited?<br>کیا آپ کو الٹی ہوئی ہے؟	1. Not at Allبالکل بھی نہیں<br>2. A littleتھوڑی سی<br>3. Quite a Bitکافی حد تک<br>4.Very Much بہت زیادہ
17.	Have you been constipated?<br>کیا آپ کو قبض کی شکایت ہوئی ہے؟	1. Not at Allبالکل بھی نہیں<br>2. A littleتھوڑی سی<br>3. Quite a Bitکافی حد تک<br>4.Very Much بہت زیادہ
18.	Have you had diarrhea?<br>کیا آپ کو دست / موشن ہوئے؟	1. Not at Allبالکل بھی نہیں<br>2. A littleتھوڑی سی<br>3. Quite a Bitکافی حد تک<br>4.Very Much بہت زیادہ
19.	Were you tired?<br>کیا درد کی وجہ سے آپ کی روزمرّہ کی زندگی متاثر ہوئی؟	1. Not at Allبالکل بھی نہیں<br>2. A littleتھوڑی سی<br>3. Quite a Bitکافی حد تک<br>4.Very Much بہت زیادہ
20.	Have you had difficulty in concentrating on things, like reading a newspaper or watching television?<br>کیا آپ کو چیزوں پر توجّہ مرکوز کرنے میں مشکل پیش آئی ہے، جیسے کہ اخبار پڑھنا یا ٹیلیویژن دیکھنا؟	1. Not at Allبالکل بھی نہیں<br>2. A littleتھوڑی سی<br>3. Quite a Bitکافی حد تک<br>4.Very Much بہت زیادہ
21.	Did you feel tense?<br>کیا آپ نے تناؤ محسوس کیا؟	1. Not at Allبالکل بھی نہیں<br>2. A littleتھوڑی سی<br>3. Quite a Bitکافی حد تک<br>4.Very Much بہت زیادہ

22.	Did you worry?<br>کیا آپ پریشان ہوئے؟	1. Not at All بالکل بھی نہیں<br>2. A little تھوڑی سی<br>3. Quite a Bit کافی حد تک<br>4.Very Much بہت زیادہ
23.	Did you feel irritable?<br>کیا آپ نے چڑچڑا پن محسوس کیا؟	1. Not at All بالکل بھی نہیں<br>2. A little تھوڑی سی<br>3. Quite a Bit کافی حد تک<br>4.Very Much بہت زیادہ
24.	Did you feel depressed?<br>کیا آپ نے افسردگی محسوس کی؟	1. Not at All بالکل بھی نہیں<br>2. A little تھوڑی سی<br>3. Quite a Bit کافی حد تک<br>4.Very Much بہت زیادہ
25.	Have you had difficulty remembering things?<br>کیا آپ کو چیزوں کو یاد رکھنے میں مشکل پیش آئی ہے؟	1. Not at All بالکل بھی نہیں<br>2. A little تھوڑی سی<br>3. Quite a Bit کافی حد تک<br>4.Very Much بہت زیادہ
26.	Has your physical condition or medical treatment interfered with your <u>family life (family members and spouse)?</u><br>کیا آپ کی جسمانی حالت یا علاج معلجہ آپ کے اہل خانہ کی زندگی کی لیئے مشکلات کا باعث بنا؟	1. Not at All بالکل بھی نہیں<br>2. A little تھوڑی سی<br>3. Quite a Bit کافی حد تک<br>4.Very Much بہت زیادہ
27.	Has your physical condition or medical treatment interfered with your <u>social activities?</u><br>کیا آپ کی جسمانی حالت یا طبّی علاج آپ کی سماجی سرگرمیوں کی لیئے مشکلات کا باعث بنی؟	1. Not at All بالکل بھی نہیں<br>2. A little تھوڑی سی<br>3. Quite a Bit کافی حد تک<br>4.Very Much بہت زیادہ
28.	Has your physical condition or medical treatment caused you <u>financial difficulties?</u><br>کیا آپ کی جسمانی حالت علاج معلجہ نے آپ کو مالی مشکلات سے دوچار کیا ہے؟	1. Not at All بالکل بھی نہیں<br>2. A little تھوڑی سی<br>3. Quite a Bit کافی حد تک<br>4.Very Much بہت زیادہ

29.	On a scale of 1 to 7 How would you rate your overall health during the past week?<br><br>پچھلے ہفتے کے دوران آپ اپنی مجموعی صحت کو کیا درجہ دیں گی؟	1. Very poor بہت ہی خراب<br>2.<br>3.<br>4.<br>5.<br>6.<br>7. Excellent بہترین
30.	On a scale of 1 to 7 How would you rate your overall quality of life during the past week?<br>پچھلے ہفتے کے دوران آپ اپنی / اپنے معیارِ زندگی کو کیا درجہ دیں گی / گے	1. Very poor بہت ہی خراب<br>2.<br>3.<br>4.<br>5.<br>6.<br>7. Excellentبہترین

EORTC QLQ - BN20 (Brain)(دماغ)

	**During the past weekپچھلے ہفتے کے دوران**	**Response**
31.	Did you feel uncertain about the future?<br>کیا آپ نے اپنے مستقبل کے بارے میں غیر یقینی محسوس کی؟	1. Not at Allبالکل بھی نہیں<br>2. A littleتھوڑی سی<br>3. Quite a Bitکافی حد تک<br>4.Very Much بہت زیادہ
32.	Did you feel you had setbacks in your condition?<br>کیا آپ کو یہ احساس ہوا کہ آپ کی حالت میں خرابی آئی ہے؟	1. Not at Allبالکل بھی نہیں<br>2. A littleتھوڑی سی<br>3. Quite a Bitکافی حد تک<br>4.Very Much بہت زیادہ
33.	Were you concerned about disruption of family life?<br>کیا آپ اپنے گھریلو زندگی میں بگاڑ کی وجہ سے فکرمند تھے؟	1. Not at Allبالکل بھی نہیں<br>2. A littleتھوڑی سی<br>3. Quite a Bitکافی حد تک<br>4.Very Much بہت زیادہ
34.	Did you have headaches?<br>کیا آپ کے سر میں درد ہوا؟	1. Not at Allبالکل بھی نہیں<br>2. A littleتھوڑی سی<br>3. Quite a Bitکافی حد تک<br>4.Very Much بہت زیادہ

35.	Has your outlook on the future worsen?<br>کیا آپ کو محسوس ہوا کہ آپ کا مستقبل تاریک ہے؟	1. Not at Allبالکل بھی نہیں<br>2. A littleتھوڑی سی<br>3. Quite a Bitکافی حد تک<br>4.Very Much بہت زیادہ
36.	Did you have double vision?<br>کیا آپ کو ایک شکل ڈبل / دو نظر آتی؟	1. Not at Allبالکل بھی نہیں<br>2. A littleتھوڑی سی<br>3. Quite a Bitکافی حد تک<br>4.Very Much بہت زیادہ
37.	Was your vision blurred?<br>کیا آپ کو دھندلا دیکھائی دیا؟	1. Not at Allبالکل بھی نہیں<br>2. A littleتھوڑی سی<br>3. Quite a Bitکافی حد تک<br>4.Very Much بہت زیادہ
38.	Did you have difficulty reading because of your vision?<br>*Includes any kind of difficulty*<br>کیا نظر کے باعث آپ کو پڑھنے میں مشکل پیش آئی؟	1. Not at Allبالکل بھی نہیں<br>2. A littleتھوڑی سی<br>3. Quite a Bitکافی حد تک<br>4.Very Much بہت زیادہ
39.	Did you have seizures?<br>کیا آپ کو دورے پڑے؟	1. Not at Allبالکل بھی نہیں<br>2. A littleتھوڑی سی<br>3. Quite a Bitکافی حد تک<br>4.Very Much بہت زیادہ
40.	Did you have weakness on one side of your body?<br>کیا آپ کے جسم کے ایک طرف حصّہ میں کمزوری آئی؟	1. Not at Allبالکل بھی نہیں<br>2. A littleتھوڑی سی<br>3. Quite a Bitکافی حد تک<br>4.Very Much بہت زیادہ
41.	Did you have trouble finding the right words to express yourself?<br><br>***Note: Not able to identify correct word to express***<br>کیا آپ کو اپنی بات سمجھانے کے لئے موزوں الفاظ استعمال کرنے میں دشواری پیش آئی؟	1. Not at Allبالکل بھی نہیں<br>2. A littleتھوڑی سی<br>3. Quite a Bitکافی حد تک<br>4.Very Much بہت زیادہ
42.	Did you have difficulty speaking?<br>کیا آپ کو بولنے میں دشواری پیش آئی؟	1. Not at Allبالکل بھی نہیں<br>2. A littleتھوڑی سی<br>3. Quite a Bitکافی حد تک<br>4.Very Much بہت زیادہ

43.	Did you have trouble communicating your thoughts?<br>کیا آپ کو اپنے خیالات کا اظہار کرنے میں دشواری پیش آئی؟	1. Not at Allبالکل بھی نہیں<br>2. A littleتھوڑی سی<br>3. Quite a Bitکافی حد تک<br>4.Very Much بہت زیادہ
44.	Did you feel drowsy during the daytime?<br>کیادن کے اوقات میں آپ نے غنودگی محسوس کی؟	1. Not at Allبالکل بھی نہیں<br>2. A littleتھوڑی سی<br>3. Quite a Bitکافی حد تک<br>4.Very Much بہت زیادہ
45.	Did you have trouble with your coordination?<br>کیا آپ کو اپنی سرگرمیوں کی ہم آہنگی میں مشکل پیش آئی؟	1. Not at Allبالکل بھی نہیں<br>2. A littleتھوڑی سی<br>3. Quite a Bitکافی حد تک<br>4.Very Much بہت زیادہ
46.	Did hair loss bother you?<br>کیابالوں کے گرنے نے آپ کو تنگ کیا ہے؟	1. Not at Allبالکل بھی نہیں<br>2. A littleتھوڑی سی<br>3. Quite a Bitکافی حد تک<br>4.Very Much بہت زیادہ
47.	Did itching of your skin bother you?<br>کیاجلد کی خارش نے آپ کو پریشان کیا ہے؟	1. Not at Allبالکل بھی نہیں<br>2. A littleتھوڑی سی<br>3. Quite a Bitکافی حد تک<br>4.Very Much بہت زیادہ
48.	Did you have weakness of both legs?<br>کیا آپ کو دونوں ٹانگوں میں کمزوری محسوس ہوئی؟	1. Not at Allبالکل بھی نہیں<br>2. A littleتھوڑی سی<br>3. Quite a Bitکافی حد تک<br>4.Very Much بہت زیادہ
49.	Did you feel unsteady on your feet?<br>کیا آپ نے اپنے پیروں میں لڑکھڑاہٹ محسوس کی؟	1. Not at Allبالکل بھی نہیں<br>2. A littleتھوڑی سی<br>3. Quite a Bitکافی حد تک<br>4.Very Much بہت زیادہ
50.	Did you have trouble controlling your bladder or urine?<br>کیا آپ کو اپنے پیشاب پر قابو/روکنا میں دشواری پیش آئی؟	1. Not at Allبالکل بھی نہیں<br>2. A littleتھوڑی سی<br>3. Quite a Bitکافی حد تک<br>4.Very Much بہت زیادہ
