# Supplementary material for: Translation and validation of the Urdu version of the European organization for research and treatment of cancer core quality of life questionnaire (EORTC QLQ-C30) and brain module (QLQ-BN20) in primary brain tumor patients
Source: J Patient Rep Outcomes. 2021 Sep 6;5:79. doi: 10.1186/s41687-021-00354-6 (PMC8421474; doi:10.1186/s41687-021-00354-6)
Supplement: Supplementary file 2 — Additional file 2: Difficulties in Translation. [file 41687_2021_354_MOESM2_ESM.pdf]

Supplementary Material

Difficulties in Translation: The difficulty that we faced was in translating a few terminologies from English to Urdu. However, this problem was resolved by a third independent translator.

- For item 1 سخت قسم was replaced by محنت طلب.
- In item 2 تکلیف was replaced by دشواری.
- For item 19 سرگرمیاں was replaced by زندگی.
- For item 26, طبّی علاج was replaced by علاج معلجہ.
- For item 31 the word شکار was replaced by محسوس.
- For item 36 the phrase دو دو نظر was replaced by دو نظر آتی / شکل ڈیل.
- For item 37 the phrase نظر دھندلاگئی was replaced by ھندلا دیکھائی دیا.
- For item 41 الفاظ تلاش کرنے میں تکلیف ہوئی was replaced by الفاظ استعمال کرنے میں دشواری پیش آئی.
- For item 45 the words اپنی ہم آہنگی میں مشکل پیش آئی was replaced by اپنی سرگرمیوں کی ہم آہنگی میں مشکل پیش آئی.
- For item 50 the words پیشاب پر قابو پانے میں تکلیف ہوئی was replaced by

پیشاب پر قابو/ روکنا میں دشواری پیش آئی
